# 2030 In Sight: the future of global eye health

**DOI:** 10.1038/s41433-023-02815-2

**Published:** 2023-11-20

**Authors:** Jude Stern, Sumrana Yasmin, M. Babar Qureshi, Rupert Bourne

**Affiliations:** 1 Knowledge Management, International Agency for the Prevention of Blindness (IAPB), Sydney, NSW Australia; 2 Policy and Programme Strategy, Sightsavers, Islamabad, Pakistan; 3 Inclusive Eye Health, CBM, Cambridge, UK; 4 Department of Ophthalmology, Cambridge University Hospitals, Cambridge, UK; 5 Vision & Eye Research Institute, School of Medicine, Anglia Ruskin University, Cambridge, UK

**Keywords:** Public health, Outcomes research, Technology, Risk factors, Health occupations

There are 1.1 billion people around the world living with the consequences of sight loss, mostly because they do not have access to eye care services and assistive products. Most people affected by sight loss live in low- and middle-income settings with sight loss due to avoidable causes. Women and girls, aging populations and the most vulnerable people are disproportionately affected by sight loss. Without change, this will rise to 1.8 billion people by 2050 [1].

Unless there is significant investment, eye health systems are unlikely to cope with future needs.

2030 In Sight is a global strategy written and embraced by the sector drawing on the WHO World Report on Vision, the Lancet Global Health Commission on Global Eye Health and the landmark UN Resolution, Vision for Everyone. To meet the growing challenges, we need to think differently and act collectively.

By 2030, we want to see a world where:

- No one experiences unnecessary or preventable sight loss, and everyone can achieve their full potential.
- Eye care and rehabilitation services are accessible, inclusive, and affordable to everyone, everywhere, whenever they are needed.
- People understand the importance of caring for their own eye health and demand access to services free from the weight of any social stigma.

The approach to achieving the goals of 2030 In Sight centres around three key elements:

1. ELEVATE vision as a fundamental economic, social and development issue.
  Ensuring good eye health improves a person’s overall health and well-being, reduces poverty, increases education attainment and economic activity. Sight loss costs the global economy $411 billion a year in lost productivity. Good eye health is critical to achieving eight of the Sustainable Development Goals [2].
  By elevating eye health into the social, economic and development agenda, there is an opportunity to influence the widest audience possible and engage with diverse stakeholders to take responsibility for eye health such as ministries of education, finance, women development and climate action, workplaces and big business, social enterprises and economic institutions.
  Following the UN resolution on eye health in 2021, there has been a noticeable increase in the inclusion in eye health at the UN and in particular the recent release of the joint IAPB-International Labor Organisation report on Eye Health and the Workplace and UN Women Gender Equality and Eye Health reports. At a global level, these reports open discussions with new stakeholders and become useful tools to advocate for integration of eye care into broader eco-systems at the national and sub-national levels.
2. INTEGRATE eye health in wider health care systems.
  A total of 90% of sight loss is avoidable with early detection and treatment, and eye care solutions are among the most cost-effective health interventions in the world. However, there are significant inequities in the quality and coverage of eye health services, a shortage of eye health workforce, and poor integration across eye health and health systems [3].
  Integrated People-Centred Eye Care (IPEC) is one of the more effective ways we can meet the growing need and demand of eye health services globally. As a sector, we need to embrace IPEC and advocate for its adoption. We will have to come together at national and sub-national levels to drive this change and actively promote this agenda to governments. This will require national policy dialogues to develop national strategy, integration plans and policies with appropriate resource allocation for IPEC. The WHO Guide to Action is designed to assist governments and the eye health sector to work together to integrate eye health at all levels of health care [4].
  WHO has set two global eye care targets focusing on an increase in Effective Coverage for Cataract and Refractive Error by 2030 to drive action. The 2023 baseline report on effective coverage for eye care highlights the need for the global eye health sector to work collectively to address quality and equitable coverage of services.
  The workforce shortage and technological development requires us to think differently and presents opportunities to scale up eye care services in new ways to meet the goals of universal eye health coverage.
3. ACTIVATE patient, consumer and market change.
  Almost every person will have an eye health issue in their lifetime. There is a large need for patients and consumers to better understand their eye health needs and be able to demand the services and products when and where they need them. Reducing the stigma of eye care services and products is critical, particularly for girls, women, and the most vulnerable populations to be able to access and utilise eye care services. We need individuals and communities around the world to make their vision a priority and to understand the link with their wider health, grasp the social and economic impacts of inaction and take the steps needed.
  Eye care is a universal issue, and we must activate universal demand.
  By campaigning on a new level, in an innovative way and using existing campaigns like Love Your Eyes and World Sight Day, the 2030 In Sight Strategy will educate the public, governments, and world leaders to help raise the profile and awareness of the issue.
  Together, we must work to make sure eye health receives the global political, health and development focus it needs and deserves.
  In 2022, the Love Your Eyes campaign saw 20 parliament screenings, over 6.5 million pledges from consumers, 11,344 media pieces and 542 million media impressions. In 2023, the campaign will focus on eye care in the workplace. Together, we will encourage employers to make eye health initiatives standard practice and promote eye health-seeking behaviour that will benefit the well-being, safety, and productivity of millions of employees.
  To ACTIVATE market change, a large opportunity exists to utilise and harness the role of the private sector. Effective and efficient markets with affordable products and services can be a major part of the solution. There are already strong examples of good public-private partnerships including the collegial approach with the pharmaceutical sector on tackling onchocerciasis and trachoma. We can extend this concept to other areas including tackling conditions such as diabetic retinopathy, and in creating sustainable, affordable, and accessible markets for spectacles.
  To help create the right market environment, we will have to break down regulatory and financial barriers to help expand access to affordable eye health services and products.

The scale of the change that is needed and the urgency to accelerate action in the next 7 years means we have to think differently and act cohesively as a sector.

The challenges have never been greater but nor have the opportunities. As the overarching alliance for global eye health, the IAPB, via its 180+ members, provides a network for engagement, inspiration, and action to collectively achieve 2030 In Sight.
